# Retinal Arterial Wall Reflectivity and Incident Cardiovascular Disease

**DOI:** 10.1016/j.xops.2026.101399

**Published:** 2026-09-04

**Authors:** Ran Xiang, Yuki Muraoka, Takahiro Kogo, Yu Hidaka, Yuki Mori, Masayuki Hata, Masahiro Miyake, Satoshi Morita, Takahiro Horie, Koh Ono, Yasuharu Tabara, Fumihiko Matsuda, Akitaka Tsujikawa

**Affiliations:** 1 Department of Ophthalmology and Visual Sciences, Kyoto University Graduate School of Medicine, Kyoto, Japan; 2 Department of Biomedical Statistics and Bioinformatics, Kyoto University Graduate School of Medicine, Kyoto, Japan; 3 Institute for Advancement of Clinical and Translational Science, Kyoto University Hospital, Kyoto, Japan; 4 Department of Cardiovascular Medicine, Kyoto University Graduate School of Medicine, Kyoto, Japan; 5 Graduate School of Public Health, Shizuoka Graduate University of Public Health, Shizuoka, Japan; 6 Center for Genomic Medicine, Kyoto University Graduate School of Medicine, Kyoto, Japan

**Keywords:** Optical coherence tomography, Retinal arterial wall reflectivity, Cardiovascular disease, Stroke, Microvascular health

## Abstract

**Objective:**

To determine whether retinal arterial wall reflectivity is associated with future cardiovascular disease (CVD) in a large community-based Japanese cohort.

**Design:**

Community-based cohort study.

**Participants:**

A total of 6264 adults from the Nagahama Study (baseline 2012–2016) without a history of CVD.

**Methods:**

Retinal arterial wall reflectivity was measured on peripapillary OCT images at baseline. Participants were followed for approximately 7 years, and incident CVD events, including stroke and coronary artery disease (CAD), were identified through medical records. Associations were evaluated using Fine–Gray competing risk models and expressed as subdistribution hazard ratios (SHRs). Retinal arterial caliber was included in multivariable analyses.

**Main Outcome Measures:**

Incident CVD events, including stroke and CAD.

**Results:**

During a mean follow-up of 6.7 years, 125 incident CVD events occurred. In the full cohort, retinal arterial wall reflectivity was not independently associated with CVD (SHR per 10 arbitrary units, 1.026; 95% confidence interval [CI], 0.920–1.145). A continuous age-by-reflectivity interaction was observed (*P* = 0.029). In post hoc exploratory analyses among participants younger than 70 years, parsimonious models suggested possible associations with CVD (SHR, 1.173; 95% CI, 1.031–1.334) and stroke (SHR, 1.280; 95% CI, 1.088–1.506).

**Conclusions:**

Retinal arterial wall reflectivity was not independently associated with incident CVD in the full cohort. Post hoc exploratory analyses among participants younger than 70 years suggested possible modest associations with CVD, particularly stroke-related outcomes. These findings suggest that retinal arterial wall reflectivity may reflect subclinical vascular alterations, but require external validation.

**Financial Disclosure(s):**

Proprietary or commercial disclosure may be found in the Footnotes and Disclosures at the end of this article.

Cardiovascular disease (CVD), including stroke and coronary artery disease (CAD), remains a leading cause of morbidity and mortality worldwide.^1^^,^^2^ Current cardiovascular risk assessment models primarily rely on demographic and systemic clinical parameters, including age, sex, blood pressure, lipid profiles, smoking status, and diabetes.^3^^,^^4^ In addition to conventional cardiovascular risk factors, arterial stiffness assessed by brachial-ankle pulse wave velocity (baPWV) is an established marker of macrovascular aging and cardiovascular risk.^5^, ^6^, ^7^ However, macrovascular stiffening and microvascular alterations do not always occur in parallel.^8^ Therefore, investigating retinal microvascular alterations may provide additional insight into vascular changes not fully captured by systemic macrovascular markers.^9^

The retinal vasculature offers a unique opportunity for noninvasive evaluation of the systemic microcirculation.^10^^,^^11^ As an accessible neurovascular tissue, the retina allows direct visualization of small vessels that share anatomical and physiological characteristics with the cerebral microcirculation.^12^ Previous epidemiological studies, including the Atherosclerosis Risk in Communities Study,^13^ Rotterdam Study,^14^ and Multi-Ethnic Study of Atherosclerosis,^15^ demonstrated that retinal vascular abnormalities assessed using fundus photography are associated with stroke and systemic vascular disease. In particular, a narrower retinal arterial caliber, quantified as the central retinal arteriolar equivalent (CRAE), has been consistently associated with an increased risk of incident stroke, potentially reflecting shared cerebrovascular pathology.^11^^,^^15^ However, conventional fundus photography is limited to a two-dimensional assessment of vascular caliber and cannot directly evaluate the retinal vascular wall structure.

OCT addresses these limitations by providing high-resolution cross-sectional imaging of retinal tissues, enabling quantitative assessment of retinal vascular wall structure.^16^^,^^17^ In prior cross-sectional analyses using OCT-based retinal vascular imaging, we demonstrated that retinal arterial wall reflectivity was significantly associated with aging and systemic vascular risk factors such as blood pressure, arterial stiffness, and sleep-related risk factors.^9^^,^^16^^,^^18^ Beyond retinal vascular caliber assessed using fundus photography, arterial wall reflectivity assessed using OCT may reflect microstructural vascular alterations not directly captured by conventional caliber-based retinal vascular assessment.^9^^,^^19^

Despite growing interest in OCT-based retinal vascular imaging, the association between OCT-detected retinal microvascular structural alterations and the subsequent development of incident CVD events remains unclear. Examining this longitudinal relationship may help clarify whether OCT-based retinal vascular wall imaging captures vascular information beyond that provided by conventional retinal vascular caliber assessment. In this longitudinal analysis of a large community-based Japanese cohort, we investigated the association between OCT-derived retinal vascular structural parameters and incident CVD events, with a particular focus on arterial wall reflectivity as a potential imaging marker of cardiovascular risk.

## Methods

### Study Population and Data Collection

This longitudinal study analyzed data from the Nagahama Prospective Cohort for Comprehensive Human Bioscience, a large community-based cohort of approximately 10 000 community-dwelling residents of Nagahama City, Japan, established to investigate environmental and genetic determinants of health. Baseline ophthalmic examinations for the present analysis were conducted during the second survey cycle between 2012 and 2016. The study protocol adhered to the tenets of the Declaration of Helsinki and was approved by the Institutional Review Board of Kyoto University Graduate School of Medicine and the Nagahama Municipal Review Board. Written informed consent was obtained from all participants prior to their enrollment.

To ensure reliable retinal vascular assessment and accurate outcome ascertainment, participants were excluded sequentially for the following reasons: history of ocular surgery other than cataract surgery; high myopia (axial length >26.0 mm); hyperopia (axial length <21.0 mm); major retinal or optic nerve disease; history of CVD at baseline; pregnancy; incomplete or inadequate ocular imaging data, including unavailable or ungradable right-eye images; and missing key systemic information.

### Systemic and Ophthalmic Assessments

Systemic and ophthalmic parameters were measured using standardized protocols. Information on lifestyle factors and medical history was obtained using standardized self-administered questionnaires. Smoking history was defined as current or previous smoking. Diabetes was defined as a physician-diagnosed diabetes history. Blood pressure was measured twice using an automated oscillometric device after at least 5 minutes of seated rest, and the average of the two measurements was used for analysis. Systemic arterial stiffness was evaluated by measuring the baPWV. Following a resting period in the supine position, arterial waveforms and blood pressure were simultaneously recorded at the brachia and ankle using an automated device (Vasera-1500; Fukuda Denshi Co, Ltd). The baPWV was calculated from the time interval between the wave fronts of the brachial and ankle arterial waveforms and the estimated anatomical path length based on the participant's height.^7^

Fasting blood and spot urine samples were obtained from all participants. Total cholesterol was determined using the cholesterol dehydrogenase method (Determiner C-TC; Kyowa Medix), and high-density lipoprotein cholesterol (HDL-C) was measured using a direct method (Determiner L HDL-C; Kyowa Medix) on an automated biochemical analyzer (AU-5431; Beckman Coulter). Non–HDL C was calculated as total cholesterol minus HDL-C. Urinary protein was assessed using urine dipstick testing (Uropaper αIII; Eiken Chemical) on an automated urine analyzer (US-2100; Eiken Chemical).

Ophthalmic examinations included right-eye fundus photography using a 45-degree digital nonmydriatic camera (CR-DGi; Canon) and spectral-domain OCT (RS-3000 Advance; NIDEK). Retinal vascular caliber was analyzed from right-eye fundus photographs using a semiautomated computer-assisted program (IVAN; University of Wisconsin), and the CRAE was calculated using established formulas with adjustment for ocular magnification.^7^

### OCT-Based Vessel Assessments

Retinal vessel parameters were assessed using right-eye peripapillary circular B-scans (3.45 mm in diameter) centered on the optic disc (Fig 1), following a standardized protocol previously used in the Nagahama cohort.^16^ Right-eye images were used for all participants to maintain consistent laterality. Within the right eye, all identifiable retinal arteries were reviewed, and the four largest measurable arteries were selected according to outer diameter.

**Figure 1:**
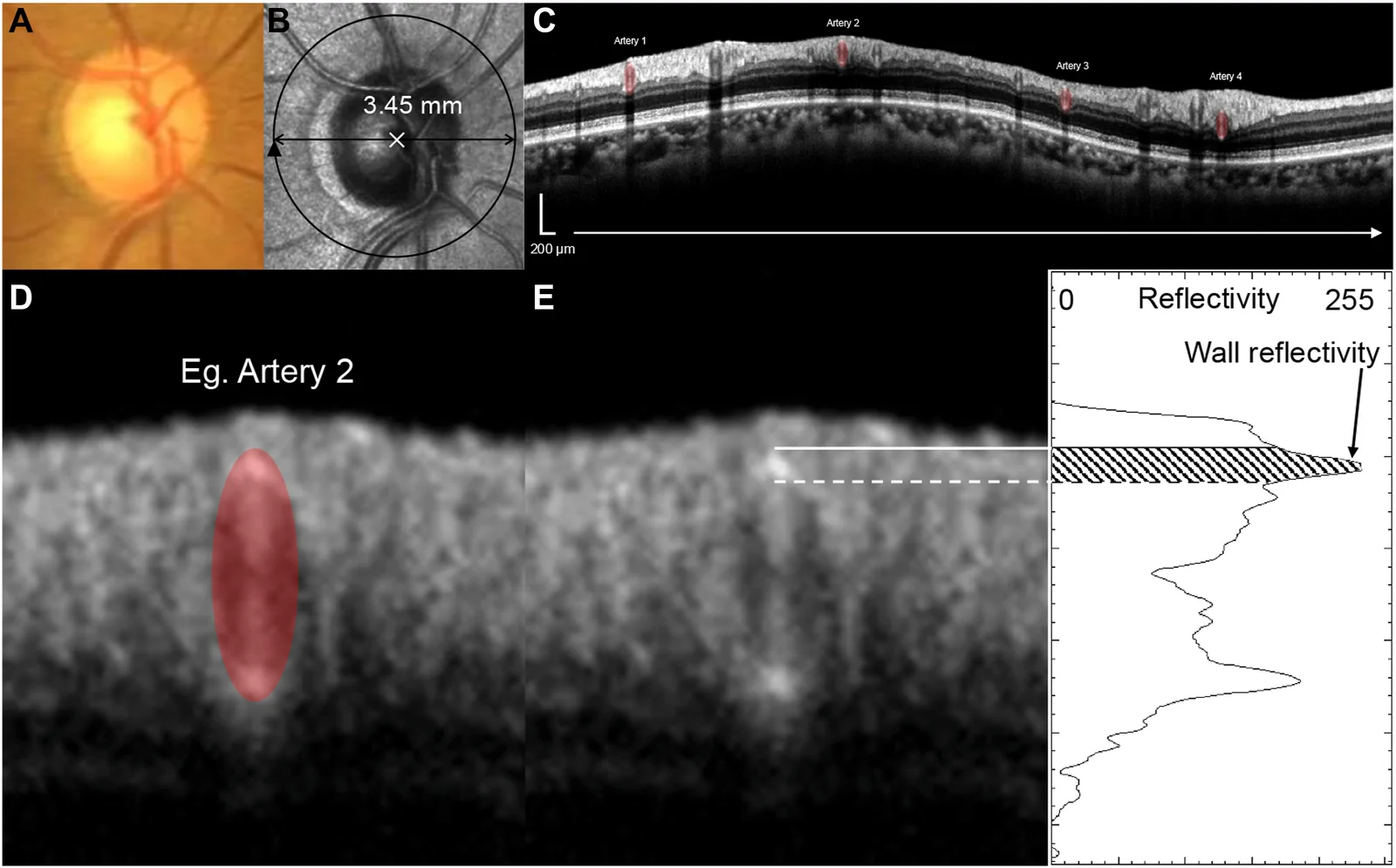
Quantification of retinal arterial parameters using OCT. (**A**) Color fundus photograph. (**B**) Scanning laser ophthalmoscopy image. The white circular arrow represents the location and direction of the circular OCT scan around the optic disc, which was used to acquire cross-sectional images of the retinal vessels. (**C**) Circular OCT image around the optic disc. A representative OCT B-scan image shows cross-sectional views of the retinal vessels. Red ovals indicate arteries. (**D**) Magnified images of retinal arterial cross-section. (**E**) Representative plot profile used to assess arterial wall reflectivity.

As described in prior investigations,^16^ four arterial parameters were quantified from OCT images: outer diameter, inner diameter, wall thickness (defined as half the difference between the outer and inner diameters), and wall reflectivity. The wall reflectivity was measured within the inner vessel wall to minimize the signal attenuation from intraluminal blood.

To account for interindividual variability in OCT signal intensity, the wall reflectivity was normalized using reflectivity values from the vitreous body and the retinal nerve fiber layer (RNFL),^20^^,^^21^ according to the following formula:

$$\text{Wall}\;\text{reflectivity}=\frac{\left(\text{Raw}\;\text{wall}\;\text{reflectivity}–\text{Vitreous}\;\text{body}\;\text{reflectivity}\right)}{\left(\text{RNFL}\;\text{reflectivity}–\text{Vitreous}\;\text{body}\;\text{reflectivity}\right)}\times 100$$

For each vessel, the wall reflectivity was measured along a line perpendicular to the vessel center and along two additional parallel lines located 300 μm on either side. The raw wall reflectivity was defined as the mean value from the central line, whereas the reference reflectivity values were averaged from the two outer lines.

For each participant, the final OCT-derived arterial parameters were calculated as the mean of measurements from the four largest measurable right-eye arteries selected according to outer diameter.

All images were acquired using the same OCT device and standardized scanning protocol and were manually reviewed by trained graders blinded to participants’ clinical information. Images were excluded when poor image quality, motion artifacts, media opacity, segmentation errors, or unclear vessel-wall boundaries precluded reliable measurement. Reflectivity was normalized to the vitreous body and RNFL to reduce interscan signal-intensity variation. Quantitative OCT signal-strength values were not available in the analytic data set. The present analysis used the same image-acquisition, vessel-selection, measurement, normalization, and quality-control procedures as the previously validated protocol.^16^ In that validation sample, intraobserver and interobserver reproducibility was assessed by repeated measurements of 100 and 50 participants, respectively, and the corresponding intraclass correlation coefficients were calculated.

### Ascertainment of Cardiovascular Outcomes

Incident CVD events were ascertained through the Nagahama Study medical-record survey during up to 7 years of follow-up, following previously reported procedures.^5^ The composite endpoint was defined as the first occurrence of stroke or CAD. Clinical information was obtained from participants’ medical records held by the three emergency care hospitals in Nagahama City: Nagahama City Hospital, Nagahama Red Cross Hospital, and Nagahama City Kohoku Hospital. Trained nurses and laboratory technicians routinely visited these hospitals to identify suspected CVD cases and retrieve the records required for diagnosis. When information from hospitals outside this network was needed, those hospitals were asked to provide the relevant records. Death records maintained by the local health care center were also reviewed to identify fatal myocardial infarction or stroke. All suspected events were adjudicated by an independent physician with expertise in CVD epidemiology using the available clinical information. Whether the adjudicator was blinded to retinal OCT measurements could not be confirmed from the available documentation.

Stroke was defined as the sudden onset of neurological symptoms lasting for at least 24 hours, including death associated with neurological deficits, according to the World Health Organization Monitoring Trends and Determinants in Cardiovascular Disease project, and included cerebral infarction, intracerebral hemorrhage, and subarachnoid hemorrhage. Coronary artery disease events were defined as definite or probable myocardial infarction according to the World Health Organization Monitoring Trends and Determinants in Cardiovascular Disease criteria or coronary revascularization, including percutaneous coronary intervention/angioplasty or coronary artery bypass grafting. Myocardial infarction was diagnosed based on symptoms at onset, electrocardiographic findings, cardiac biomarkers, including creatine kinase, and coronary angiographic findings.

Follow-up time was calculated from the date of baseline OCT measurement to the first CVD event, non-CVD death, relocation from the study area, or the last confirmed date of residence, whichever occurred first. Participants were censored at the date of relocation from the study area or at the last confirmed date of residence. Death from non-CVD causes was considered a competing risk in competing-risk analyses. In outcome-specific competing-risk analyses, incident stroke was treated as the event of interest, while CAD and non-CVD death were considered competing events. Conversely, in analyses of CAD, incident CAD was treated as the event of interest, while stroke and non-CVD death were treated as competing events.

### Statistical Analyses

Baseline characteristics were summarized as means with standard deviations for continuous variables and as frequencies with percentages for categorical variables. The cumulative incidence of CVD was estimated using cumulative incidence function curves, with participants categorized into tertiles of retinal arterial wall reflectivity for visual comparison.

Associations between retinal arterial wall reflectivity and incident CVD were evaluated using Fine–Gray competing-risk regression models, with non-CVD death treated as a competing event. The primary analysis was conducted in the full cohort using a multivariable model adjusted for age, sex, systolic blood pressure, non–HDL-C, HDL-C, urinary protein, smoking history, diabetes, antihypertensive medication use, baPWV, and CRAE. Effect modification by age was evaluated in the Fine–Gray model by including an interaction term between age and retinal arterial wall reflectivity. Both variables were mean-centered before constructing the interaction term, and wall reflectivity was scaled per 10 arbitrary units (AU) increase. Restricted cubic spline analysis was used to examine whether the association between wall reflectivity and incident CVD varied nonlinearly with age. The age cutoff of 70 years was selected post hoc from the spline pattern; therefore, all age-stratified analyses were designated exploratory. To reduce the risk of model overfitting in the exploratory analysis among participants younger than 70 years, the main model was restricted to age, sex, systolic blood pressure, CRAE, and wall reflectivity. The fully adjusted model was conducted as a sensitivity analysis. Subdistribution hazard ratios (SHRs) were expressed per 10-AU increase in wall reflectivity, per 1-μm increase in CRAE, and per 1-cm/ms increase in baPWV. To assess potential selection bias, baseline characteristics were compared between participants included in and excluded from the final analysis using standardized mean differences and variable-specific denominators.

All statistical analyses were performed using R software, version 4.5.2 (R Foundation for Statistical Computing).

## Results

### Participants’ Baseline Characteristics

Of the 9850 participants who underwent examinations, 3586 were excluded, leaving 6264 participants for the final analysis (Fig 2). The final analytical cohort had a mean age of 57.2 ± 12.0 years, 30.0% were male, and mean retinal arterial wall reflectivity was 137.3 ± 13.9 AU (Table 1). Compared with participants included in the final analysis, excluded participants were older (59.5 vs. 57.2 years), more frequently male (37.0% vs. 30.0%), and had higher systolic blood pressure (126.4 vs. 123.5 mmHg), baPWV (1.33 vs. 1.27 cm/ms), diabetes prevalence (8.2% vs. 6.0%), and antihypertensive medication use (27.7% vs. 21.1%); CRAE was smaller among excluded participants with available measurements (131.8 vs. 135.4 μm), and excluded participants had higher arterial wall reflectivity (140.8 vs. 137.3 AU) (Supplementary Table S1, available at www.ophthalmologyscience.org).

**Figure 2:**
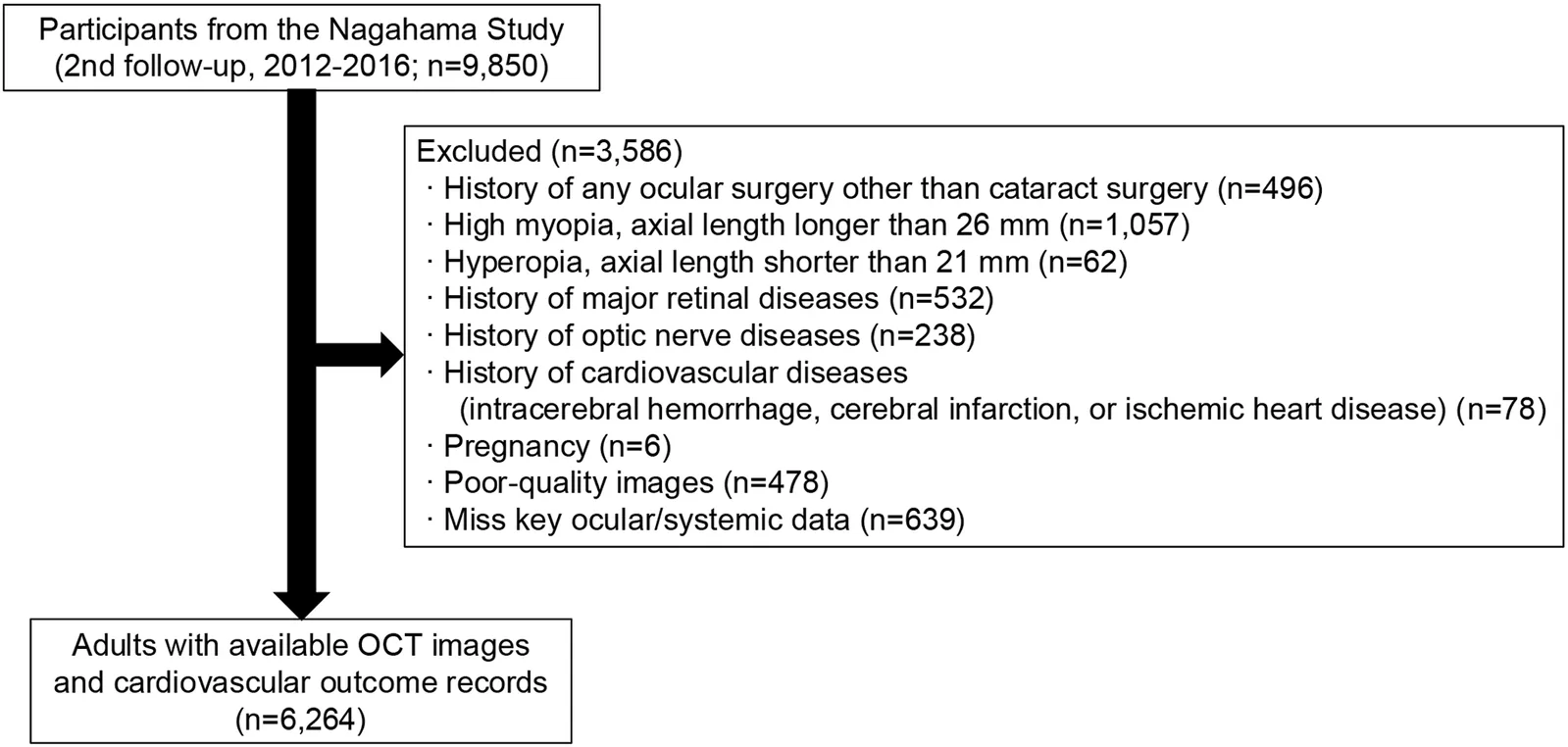
Flow diagram of participants. Flow diagram showing sequential participant selection. Of the 9850 participants who underwent examinations, 3586 were excluded from the final complete-case analysis, leaving 6264 participants for the primary analysis.

**Table 1:** Baseline Characteristics of the Participants (*n* = 6264)

Age, Years	57.2 ± 12.0
Sex, male n (%)	1878 (30.0)
Systolic blood pressure, mmHg	123.5 ± 17.6
Non-high-density lipoprotein cholesterol, mg/dL	137.30 ± 33.07
High-density lipoprotein cholesterol, mg/dL	68.24 ± 17.39
Urinary protein, 1+ or more n (%)	116 (1.9)
Smoking history, n (%)	1958 (31.3)
Diabetes, n (%)	373 (6.0)
Antihypertensive medicine use, n (%)	1321 (21.1)
baPWV, cm/ms	1.27 ± 0.27
Central retinal arteriolar equivalent, μm	135.41 ± 12.45
OCT-derived retinal arterial parameter	
Outer diameter, μm	117.39 ± 8.83
Inner diameter, μm	89.37 ± 8.10
Wall thickness, μm	14.01 ± 1.41
Wall reflectivity, AU	137.30 ± 13.93
Cardiovascular disease, n (%)	125 (2.0)
Stroke, n (%)	61 (1.0)
Coronary artery disease, n (%)	64 (1.0)

baPWV = brachial-ankle pulse wave velocity; AU = arbitrary units.

Values are presented as mean ± standard deviation for continuous variables and frequency (percentage) for categorical variables.

During a mean follow-up period of 6.7 years, 125 first incident CVD events (including 61 strokes and 64 CAD events) were recorded. Non-CVD deaths occurred in 111 participants and were treated as competing events. Participants who developed CVD were significantly older (67.7 vs. 57.0 years), had higher systolic blood pressure (137.0 vs. 123.2 mmHg), and had higher rates of smoking (54.4% vs. 30.8%) and diabetes (18.4% vs. 5.7%) than those who did not develop CVD (Supplementary Table S2, available at www.ophthalmologyscience.org).

In the prior protocol-validation sample using the same standardized OCT measurement protocol, reproducibility was good to excellent, with intraclass correlation coefficients ranging from 0.767 to 0.841 for intraobserver and from 0.815 to 0.917 for interobserver reproducibility of arterial parameters (Supplementary Table S3, available at www.ophthalmologyscience.org).

### Cumulative Incidence of CVD

The cumulative incidence of CVD events was evaluated using cumulative incidence function curves, with participants categorized into tertiles based on their retinal arterial wall reflectivity (Fig 3). The groups were defined as T1 (lowest, range: 97.50–130.27), T2 (medium, range: 130.27–140.79), and T3 (highest, range: 140.79–254.70), with each tertile comprising 2088 participants. Among the participants, the highest (T3) and medium (T2) reflectivity groups tended to show a greater cumulative incidence of CVD than the lowest (T1) reflectivity group.

**Figure 3:**
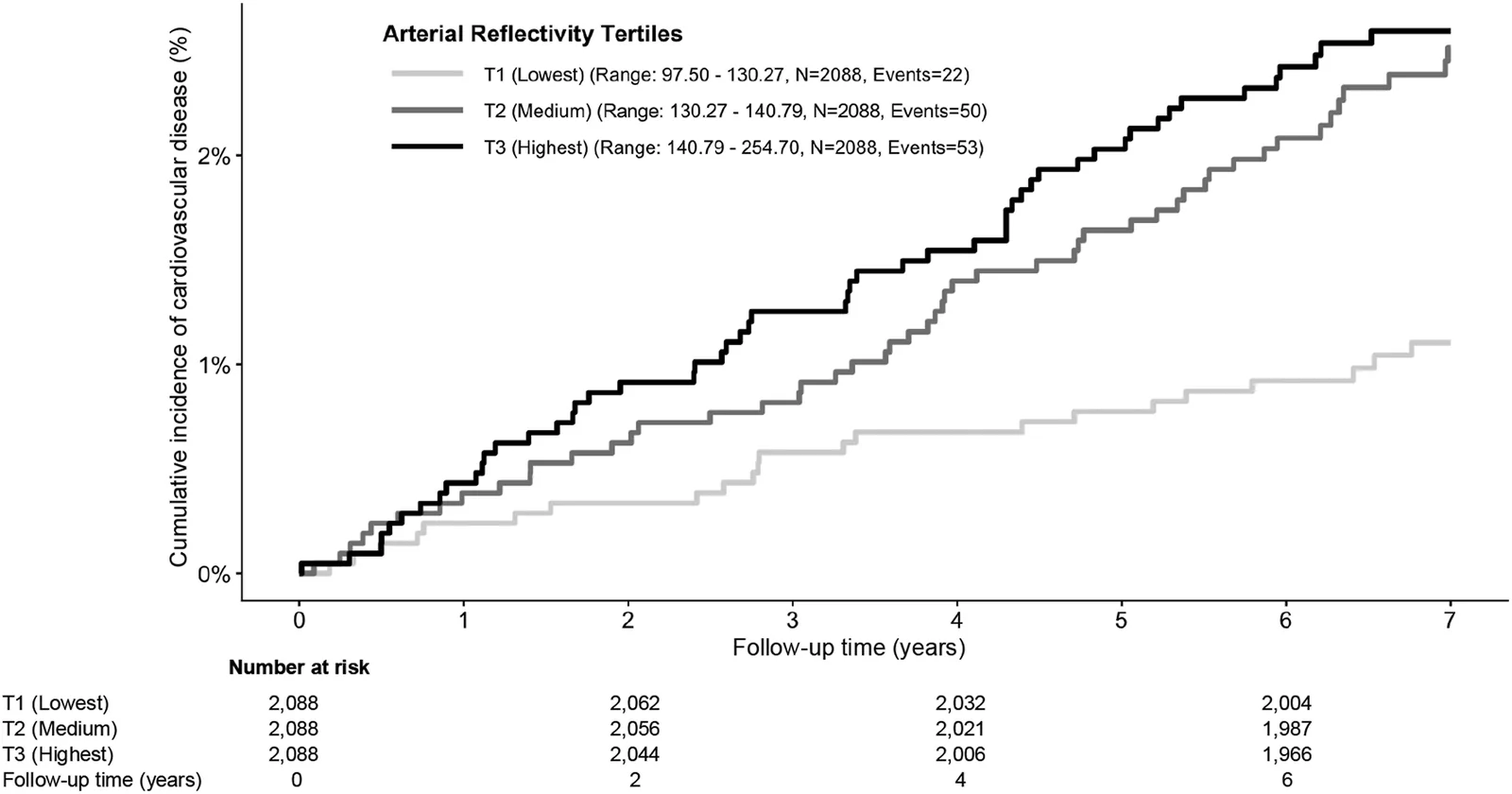
Cumulative incidence of cardiovascular disease. According to retinal arterial wall reflectivity, participants were categorized into tertiles according to their baseline retinal arterial wall reflectivity (high, medium, and low). Cumulative incidence was estimated using competing risk methods accounting for death as a competing event.

### Overall Risk Assessment

In the univariable Fine–Gray competing risk analysis, higher wall reflectivity was associated with CVD and stroke (Supplementary Table S4, available at www.ophthalmologyscience.org). These associations were attenuated in the primary full-cohort multivariable analysis (CVD: SHR per 10 AU, 1.026; 95% confidence interval [CI], 0.920–1.145; stroke: SHR per 10 AU, 1.090; 95% CI, 0.947–1.255) (Table 2). A larger CRAE was associated with lower risk of CVD (SHR per 1 μm, 0.985; 95% CI, 0.971–0.999) and stroke (SHR per 1 μm, 0.975; 95% CI, 0.957–0.993), whereas higher baPWV was associated with an increased risk of CAD (SHR per 1 cm/ms, 1.811; 95% CI, 1.405–2.333) but not stroke.

**Table 2:** Multivariable Fine–Gray Competing Risk Analysis for Cardiovascular Disease in Entire Population (n = 6264)

Variable	Cardiovascular Disease (Event = 125, Competing = 111)		Stroke (Event = 61, Competing = 175)		Coronary Artery Disease (Event = 64, Competing = 172)	
	SHR (95% CI)	*P* Value	SHR (95% CI)	*P* Value	SHR (95% CI)	*P* Value
Age, per year	1.065 (1.040–1.089)	<0.001	1.068 (1.035–1.102)	<0.001	1.064 (1.028–1.101)	<0.001
Female sex (vs. male)	0.587 (0.327–1.052)	0.074	0.678 (0.261–1.765)	0.430	0.511 (0.251–1.041)	0.064
Systolic blood pressure, per 1 mmHg	1.015 (1.003–1.026)	0.010	1.021 (1.004–1.038)	0.014	1.011 (0.997–1.025)	0.120
Non-high-density lipoprotein cholesterol, per 1 mg/dL	0.998 (0.992–1.004)	0.520	0.999 (0.991–1.008)	0.900	0.997 (0.988–1.006)	0.490
High-density lipoprotein cholesterol, per 1 mg/dL	0.986 (0.974–0.998)	0.024	0.994 (0.977–1.011)	0.470	0.978 (0.960–0.996)	0.017
Urinary protein ≥1+ (vs. < 1+)	1.771 (0.847–3.703)	0.130	2.643 (1.002–6.970)	0.050	1.185 (0.405–3.465)	0.760
Smoking history (yes vs. no)	1.532 (0.889–2.639)	0.120	1.706 (0.675–4.311)	0.260	1.407 (0.762–2.599)	0.280
Diabetes (yes vs. no)	1.490 (0.921–2.409)	0.100	0.652 (0.243–1.749)	0.400	2.402 (1.369–4.216)	0.002
No antihypertensive medication use (vs. use)	0.886 (0.607–1.293)	0.530	1.361 (0.770–2.403)	0.290	0.583 (0.344–0.990)	0.046
baPWV, per 1 cm/ms	1.432 (0.973–2.108)	0.069	0.807 (0.460–1.416)	0.450	1.811 (1.405–2.333)	<0.001
CRAE, per 1 μm	0.985 (0.971–0.999)	0.030	0.975 (0.957–0.993)	0.008	0.995 (0.976–1.014)	0.590
Arterial wall reflectivity, per 10 AU	1.026 (0.920–1.145)	0.640	1.090 (0.947–1.255)	0.230	0.981 (0.832–1.156)	0.820

SHR = subdistribution hazard ratio; CI = confidence interval; CRAE = central retinal arteriolar equivalent; baPWV = brachial-ankle pulse wave velocity; AU = arbitrary units. Reflectivity is per 10 AU, CRAE per 1 μm, and baPWV per 1 cm/ms; models include all listed variables.

### Age-Dependent Effect Modification

Restricted cubic spline analysis with four knots placed at the fifth, 35th, 65th, and 95th percentiles of the age distribution suggested a nonlinear relationship between age and the effect of reflectivity on CVD risk (P for nonlinearity = 0.046). The association between wall reflectivity and CVD risk attenuated with increasing age and approached unity around 70 years (Fig 4).

**Figure 4:**
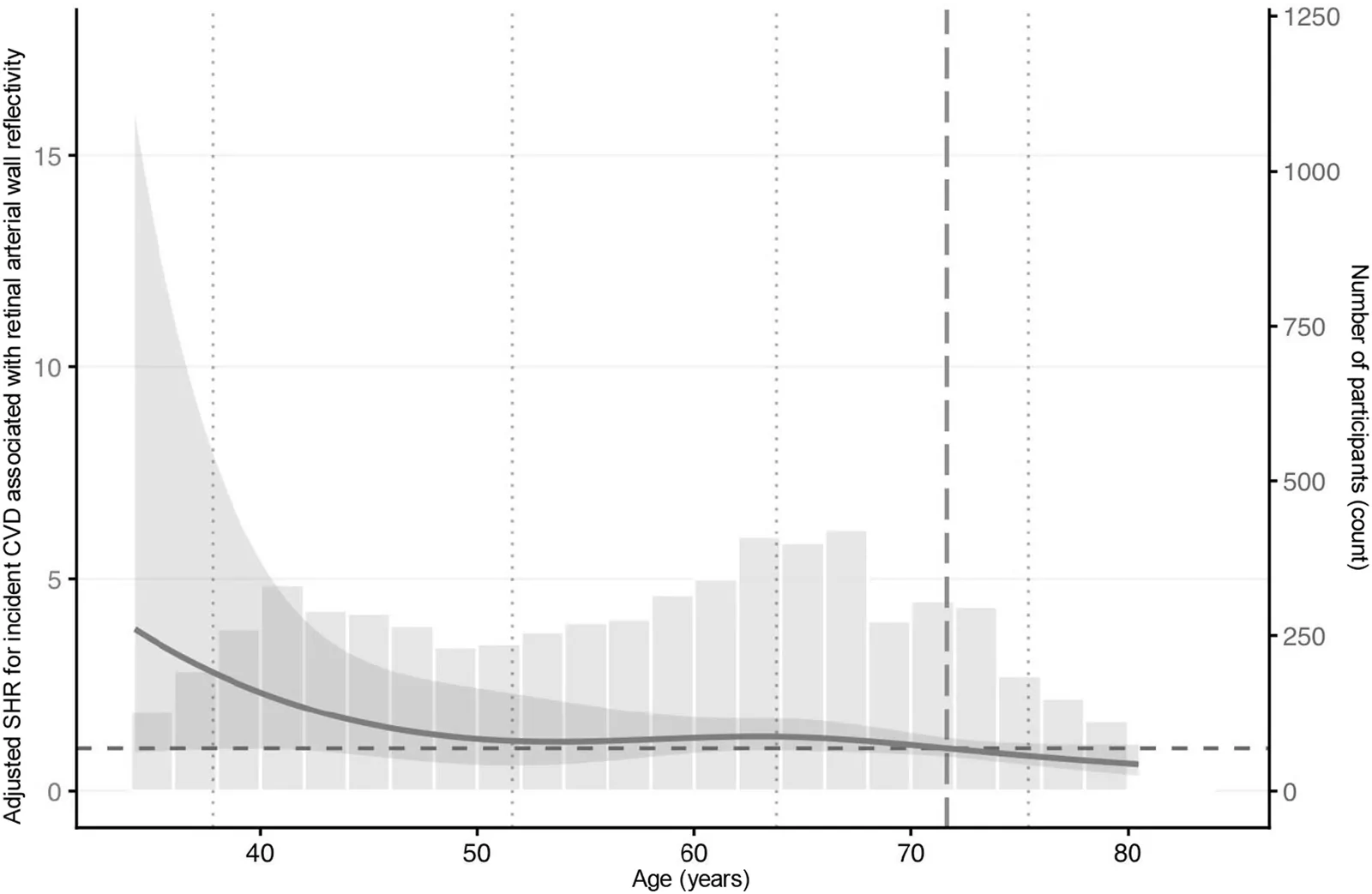
Exploratory age-dependent association of retinal arterial wall reflectivity with CVD risk. Restricted cubic spline analysis illustrating the exploratory age-dependent association between retinal arterial wall reflectivity and incident CVD. The solid line represents the adjusted SHR per 10-AU increase in reflectivity, and the shaded area indicates the 95% confidence interval. The gray histogram represents the age distribution (right y-axis). The age threshold of 70 years was selected post hoc from this pattern and requires external validation. CVD = cardiovascular disease; SHR = subdistribution hazard ratio.

In the continuous multivariable interaction model, the association between higher wall reflectivity and CVD was positive at the mean age (SHR per 10 AU, 1.215; 95% CI, 1.056–1.397) and weakened with increasing age (likelihood-ratio statistic, 4.79; *P* = 0.029; age-by-reflectivity interaction SHR, 0.985; 95% CI, 0.975–0.995; *P* = 0.004).

### Age-Stratified Effect Modification

Based on the post hoc age-dependent pattern, exploratory analyses were performed for participants younger than 70 years (n = 5207; 64 CVD events). In the parsimonious model, including age, sex, systolic blood pressure, CRAE, and wall reflectivity, higher reflectivity suggested possible associations with CVD (SHR per 10 AU, 1.173; 95% CI, 1.031–1.334) and stroke (SHR, 1.280 per 10 AU; 95% CI, 1.088–1.506), but not CAD (SHR, 1.039 per 10 AU; 95% CI, 0.874–1.236) (Table 3). A larger CRAE was associated with lower CVD and stroke risk. Fully adjusted sensitivity models yielded similar reflectivity estimates (Supplementary Table S5, available at www.ophthalmologyscience.org). No positive association was observed among participants aged 70 years or older (Supplementary Table S6, available at www.ophthalmologyscience.org).

**Table 3:** Parsimonious Fine–Gray Competing-Risk Analysis in Participants Younger than 70 Years (n = 5207)

Variable	Cardiovascular Disease (Event = 64, Competing = 54)		Stroke (Event = 33, Competing = 85)		Coronary Artery Disease (Event = 31, Competing = 87)	
	SHR (95% CI)	*P* Value	SHR (95% CI)	*P* Value	SHR (95% CI)	*P* Value
Age, per year	1.066 (1.030–1.103)	<0.001	1.054 (1.008–1.102)	0.021	1.080 (1.023–1.140)	0.005
Female sex (vs. male)	0.357 (0.218–0.584)	<0.001	0.602 (0.298–1.214)	0.160	0.197 (0.091–0.426)	<0.001
Systolic blood pressure, per 1 mmHg	1.018 (1.002–1.033)	0.024	1.014 (0.992–1.036)	0.220	1.021 (1.001–1.042)	0.041
CRAE, per 1 μm	0.972 (0.954–0.991)	0.004	0.963 (0.939–0.988)	0.004	0.983 (0.956–1.011)	0.220
Arterial wall reflectivity, per 10 AU	1.173 (1.031–1.334)	0.015	1.280 (1.088–1.506)	0.003	1.039 (0.874–1.236)	0.670

SHR = subdistribution hazard ratio; CRAE = central retinal arteriolar equivalent; CI = confidence interval; AU = arbitrary units. Models include age, sex, systolic blood pressure, CRAE, and arterial wall reflectivity. Analyses are exploratory because the age cutoff was selected post hoc.

## Discussion

In this large-scale longitudinal community-based cohort, retinal arterial wall reflectivity was not independently associated with incident CVD in the primary full-cohort multivariable analysis after adjustment for conventional cardiovascular risk factors,^22^ baPWV, and CRAE. Given the observed age interaction, post hoc exploratory age-stratified analyses were subsequently performed using the spline-derived cutoff of 70 years. In these exploratory analyses, higher arterial wall reflectivity showed possible associations with CVD and stroke among participants younger than 70 years, whereas no corresponding positive association was observed among participants aged 70 years or older. Because the primary full-cohort analysis was negative and the age-stratified analyses were post hoc, these findings should be considered hypothesis-generating rather than confirmatory.

Previous epidemiological studies using fundus photography have consistently demonstrated associations between retinal vascular abnormalities, particularly retinal arterial narrowing quantified by CRAE, and incident stroke.^23^, ^24^, ^25^ These landmark studies established the concept that retinal vascular imaging may reflect systemic and cerebrovascular alterations. The present exploratory findings are compatible with this framework and raise the possibility that OCT-derived retinal arterial wall reflectivity, which enables cross-sectional assessment of vascular wall structure, may capture aspects of retinal microvascular alteration not fully represented by vascular caliber alone.

Unlike fundus photography, which primarily evaluates vascular caliber in two-dimensional images, OCT enables direct cross-sectional visualization of retinal vascular wall morphology.^16^^,^^17^ In our previous cross-sectional studies, arterial wall reflectivity was associated with aging and systemic vascular characteristics, including blood pressure, arterial stiffness, and sleep-related factors.^9^^,^^18^ The present longitudinal findings raise the possibility that the relationship between retinal arterial wall reflectivity and subsequent cardiovascular events may vary according to age. This pattern appeared more evident for stroke-related outcomes than for CAD, although the subgroup findings should be interpreted cautiously because of their exploratory nature and the limited number of outcome events.

One possible interpretation of the observed age-related heterogeneity is that retinal arterial wall reflectivity may be more sensitive to early microvascular alterations before advanced systemic vascular remodeling and comorbidity become prevalent.^9^^,^^26^ In older individuals, greater heterogeneity in vascular pathology, a higher burden of systemic comorbidities, and competing noncardiovascular risks may reduce the relative contribution of any single retinal vascular marker.^27^ These interpretations should be regarded as speculative and warrant further investigation in experimental studies of the underlying biological mechanisms.

Previous studies have suggested close relationships between retinal and cerebral microvascular abnormalities.^28^^,^^29^ In this context, the possible association between retinal arterial wall reflectivity and stroke among participants younger than 70 years may be compatible with shared microvascular alterations affecting the retinal and cerebral circulations. In contrast, baPWV primarily reflects large-artery stiffness and showed stronger associations with CAD in this study. These differing patterns may be consistent with distinct contributions of microvascular and macrovascular alterations to CVD phenotypes, although mechanistic conclusions cannot be drawn from the present observational analyses.^8^^,^^30^

### Limitations

Several limitations should be acknowledged. First, the observational design precludes causal inference. Second, the 3586 participants excluded from the final analysis were older and differed in several vascular risk factors from included participants, indicating potential selection bias. Third, the number of events was limited, particularly in the post hoc age-stratified analyses; despite use of a parsimonious model, those estimates are exploratory and require external validation. Fourth, quantitative OCT signal-strength data were unavailable, so a formal image-quality sensitivity analysis based on signal strength could not be performed. Although all images underwent manual quality review and poor-quality images were excluded before analysis, residual effects of image quality, media opacity, segmentation quality, RNFL characteristics, or other OCT signal characteristics on wall reflectivity cannot be fully excluded. Fifth, this study focused on associations rather than clinical prediction model development. We did not evaluate whether OCT-derived arterial wall reflectivity improved discrimination, calibration, reclassification, or net clinical benefit beyond conventional cardiovascular risk factors and CRAE. Therefore, no conclusion can be drawn regarding its incremental clinical utility for cardiovascular risk stratification. Finally, the cohort consisted of community-dwelling Japanese adults, limiting generalizability.

## Conclusions

In this longitudinal community-based cohort study, retinal arterial wall reflectivity was not independently associated with incident CVD in the primary full-cohort analysis. A significant interaction between age and arterial wall reflectivity prompted post hoc exploratory analyses, which suggested possible associations with CVD and stroke among participants younger than 70 years. These findings are hypothesis-generating and require confirmation in independent cohorts before any clinical application can be considered.
