# Supplementary material for: Retinal Arterial Wall Reflectivity and Incident Cardiovascular Disease
Source: Ophthalmol Sci. 2026 Sep 4;6(11):101399. doi: 10.1016/j.xops.2026.101399 (PMC13671367; doi:10.1016/j.xops.2026.101399)
Supplement: Supplementary file 1 [file mmc1.pdf]

Supplementary Table 1. Comparison of Baseline Characteristics Between Included and Excluded Participants

Characteristic	Included cohort (n=6,264)	Excluded cohort (n=3,586)
Age, years	57.18 ± 12.00	59.49 ± 12.94
Male sex, n/N (%)	1,878/6,264 (30.0)	1,328/3,586 (37.0)
Systolic blood pressure, mmHg	123.46 ± 17.56	126.38 ± 18.35
Non-HDL cholesterol, mg/dL	137.30 ± 33.07	135.59 ± 32.61
HDL cholesterol, mg/dL	68.24 ± 17.39	66.88 ± 17.59
Urinary protein ≥1+, n/N (%)	116/6,264 (1.9)	111/3,496 (3.2)
Smoking history, n/N (%)	1,958/6,264 (31.3)	1,211/3,585 (33.8)
Diabetes, n/N (%)	373/6,264 (6.0)	294/3,585 (8.2)
Antihypertensive medication use, n/N (%)	1,321/6,264 (21.1)	993/3,585 (27.7)
baPWV, cm/ms	1.27 ± 0.27	1.33 ± 0.29
CRAE, μm	135.41 ± 12.45	131.80 ± 14.86
Arterial outer diameter, μm	117.39 ± 8.83	116.18 ± 10.03
Arterial inner diameter, μm	89.37 ± 8.10	88.43 ± 9.27
Arterial wall thickness, μm	14.01 ± 1.41	13.88 ± 1.51
Arterial wall reflectivity, AU	137.30 ± 13.93	140.80 ± 41.09

Values are mean ± standard deviation or n/N (%).

Abbreviations: AU, arbitrary units; baPWV, brachial-ankle pulse wave velocity; CRAE, central retinal arteriolar equivalent; HDL, high-density lipoprotein.
