# Supplementary material for: Retinal Arterial Wall Reflectivity and Incident Cardiovascular Disease
Source: Ophthalmol Sci. 2026 Sep 4;6(11):101399. doi: 10.1016/j.xops.2026.101399 (PMC13671367; doi:10.1016/j.xops.2026.101399)
Supplement: Supplementary file 2 [file mmc2.pdf]

Supplementary Table 2. Comparison of Characteristics Between Participants With and Without Incident Cardiovascular Disease

	Participants without CVD events (n=6,139)	Participants with CVD events (n=125)
Age, years	57.0 ± 12.0	67.7 ± 8.7
Sex, male n (%)	1,801 (29.3)	77 (61.6)
Systolic blood pressure, mmHg	123.2 ± 17.5	137.0 ± 17.1
Non-high-density lipoprotein cholesterol, mg/dL	137.29 ± 33.10	137.59 ± 31.51
High-density lipoprotein cholesterol, mg/dL	68.40 ± 17.39	60.15 ± 15.29
Urinary protein, 1+ or more n (%)	106 (1.7)	10 (8.0)
Smoking history, n (%)	1,890 (30.8)	68 (54.4)
Diabetes, n (%)	350 (5.7)	23 (18.4)
Anti-hypertensive medicine use, n (%)	1,264 (20.6)	57 (45.6)
Follow-up duration, years	6.69 ± 0.83	3.34 ± 2.00
baPWV, cm/ms	1.27 ± 0.25	1.55 ± 0.62
Central retinal arteriolar equivalent, μm	135.52 ± 12.43	129.80 ± 12.35
OCT-derived retinal arterial parameter		
Outer diameter, μm	117.39 ± 8.83	117.45 ± 8.45
Inner diameter, μm	89.37 ± 8.11	89.12 ± 7.64
Wall thickness, μm	14.01 ± 1.41	14.16 ± 1.47
Wall reflectivity, AU	137.22 ± 13.93	141.06 ± 13.43

Abbreviations: CVD, cardiovascular disease; baPWV, brachial-ankle pulse wave velocity; AU, arbitrary units.
Values are presented as mean ± standard deviation for continuous variables and frequency (percentage) for categorical variables.
