# Supplementary material for: Retinal Arterial Wall Reflectivity and Incident Cardiovascular Disease
Source: Ophthalmol Sci. 2026 Sep 4;6(11):101399. doi: 10.1016/j.xops.2026.101399 (PMC13671367; doi:10.1016/j.xops.2026.101399)
Supplement: Supplementary file 3 [file mmc3.pdf]

Supplementary Table 3. Intraclass correlation coefficient for OCT-measured retinal vessel parameters

	Intraobserver reproducibility (n = 100)			Interobserver reproducibility (n = 50)		
Retinal Vessel Parameters	Correlation coefficient	95% CI	*P*-value	Correlation coefficient	95% CI	*P*-value
Artery						
Outer diameter	0.818	0.740 to 0.873	<0.001	0.826	0.711 to 0.897	<0.001
Inner diameter	0.767	0.673 to 0.837	<0.001	0.815	0.695 to 0.891	<0.001
Wall reflectivity	0.841	0.773 to 0.890	<0.001	0.917	0.855 to 0.951	<0.001

Abbreviations: CI, confidence interval, OCT, optical coherence tomography
