# Supplementary material for: Retinal Arterial Wall Reflectivity and Incident Cardiovascular Disease
Source: Ophthalmol Sci. 2026 Sep 4;6(11):101399. doi: 10.1016/j.xops.2026.101399 (PMC13671367; doi:10.1016/j.xops.2026.101399)
Supplement: Supplementary file 4 [file mmc4.pdf]

Supplementary Table 4. Univariable Fine-Gray Competing-Risk Analysis for Cardiovascular Outcomes (n=6,264)

	Cardiovascular disease (event=125, competing=111)		Stroke (event=61, competing=175)		Coronary artery diseases (event=64, competing=172)	
Variable	SHR (95% CI)	P-value	SHR (95% CI)	P-value	SHR (95% CI)	P-value
Age, per year	1.097 (1.075–1.119)	<0.001	1.086 (1.057–1.117)	<0.001	1.106 (1.075–1.138)	<0.001
Female sex (vs male)	0.261 (0.182–0.374)	<0.001	0.336 (0.203–0.556)	<0.001	0.206 (0.122–0.347)	<0.001
Systolic blood pressure, per 1 mmHg	1.038 (1.031–1.046)	<0.001	1.037 (1.026–1.049)	<0.001	1.039 (1.030–1.048)	<0.001
Non-high-density lipoprotein cholesterol, per 1 mg/dL	1.000 (0.995–1.005)	0.910	1.002 (0.995–1.009)	0.550	0.999 (0.991–1.006)	0.710
High-density lipoprotein cholesterol, per 1 mg/dL	0.969 (0.957–0.981)	<0.001	0.980 (0.966–0.995)	0.008	0.957 (0.939–0.976)	<0.001
Urinary protein ≥1+ (vs <1+)	4.745 (2.491–9.038)	<0.001	4.786 (1.923–11.915)	<0.001	4.553 (1.827–11.343)	0.001
Smoking history (yes vs no)	2.669 (1.877–3.795)	<0.001	2.298 (1.392–3.795)	0.001	3.053 (1.858–5.016)	<0.001
Diabetes (yes vs no)	3.644 (2.318–5.730)	<0.001	1.411 (0.565–3.520)	0.460	6.316 (3.663–10.892)	<0.001
No antihypertensive medication use (vs use)	0.320 (0.225–0.455)	<0.001	0.516 (0.304–0.876)	0.014	0.210 (0.128–0.343)	<0.001
baPWV, per 1 cm/ms	2.450 (1.997–3.007)	<0.001	2.142 (1.636–2.805)	<0.001	2.592 (2.157–3.115)	<0.001
CRAE, per 1 µm	0.967 (0.955–0.978)	<0.001	0.960 (0.944–0.977)	<0.001	0.974 (0.958–0.990)	0.002
Arterial wall reflectivity, per 10 AU	1.176 (1.079–1.281)	<0.001	1.200 (1.070–1.346)	0.002	1.150 (1.013–1.305)	0.031

Abbreviations: SHR, subdistribution hazard ratio; CI, confidence interval; baPWV, brachial-ankle pulse wave velocity; AU, arbitrary units. Each row represents a separate univariable model. SHRs are reported per 10-AU increase in reflectivity, per 1-µm increase in CRAE, and per 1-cm/ms increase in baPWV.
