# Supplementary material for: Retinal Arterial Wall Reflectivity and Incident Cardiovascular Disease
Source: Ophthalmol Sci. 2026 Sep 4;6(11):101399. doi: 10.1016/j.xops.2026.101399 (PMC13671367; doi:10.1016/j.xops.2026.101399)
Supplement: Supplementary file 5 [file mmc5.pdf]

Supplementary Table 5. Fully Adjusted Sensitivity Analysis in Participants Aged <70 Years (n=5,207)

	Cardiovascular disease (event=64, competing=54)		Stroke (event=33, competing=85)		CAD (event=31, competing=87)	
**Variable**	**SHR (95% CI)**	**P-value**	**SHR (95% CI)**	**P-value**	**SHR (95% CI)**	**P-value**
Age, per year	1.053 (1.017–1.090)	0.004	1.056 (1.009–1.105)	0.020	1.054 (0.999–1.111)	0.053
Female sex (vs male)	0.746 (0.340–1.636)	0.460	1.473 (0.475–4.569)	0.500	0.337 (0.123–0.928)	0.035
Systolic blood pressure, per 1 mmHg	1.010 (0.993–1.028)	0.260	1.011 (0.987–1.035)	0.360	1.010 (0.987–1.033)	0.410
Non-high-density lipoprotein cholesterol, per 1 mg/dL	1.004 (0.997–1.011)	0.300	1.002 (0.991–1.013)	0.700	1.006 (0.997–1.015)	0.200
High-density lipoprotein cholesterol, per 1 mg/dL	0.984 (0.967–1.002)	0.075	0.992 (0.971–1.014)	0.480	0.972 (0.942–1.004)	0.086
Urinary protein ≥1+ (vs <1+)	0.220 (0.013–3.583)	0.290	1.149 (0.119–11.041)	0.900	—	—
Smoking history (yes vs no)	2.367 (1.082–5.174)	0.031	3.492 (1.168–10.440)	0.025	1.520 (0.603–3.831)	0.380
Diabetes (yes vs no)	1.361 (0.675–2.745)	0.390	0.538 (0.124–2.334)	0.410	2.273 (1.013–5.098)	0.046
No antihypertensive medication use (vs use)	0.719 (0.423–1.223)	0.220	0.955 (0.415–2.197)	0.910	0.561 (0.278–1.132)	0.110
baPWV, per 1 cm/ms	2.143 (0.902–5.090)	0.084	1.402 (0.645–3.047)	0.390	3.659 (1.446–9.262)	0.006
CRAE, per 1 μm	0.974 (0.956–0.992)	0.006	0.964 (0.940–0.988)	0.004	0.984 (0.957–1.012)	0.260
Arterial wall reflectivity, per 10 AU	1.166 (1.024–1.329)	0.021	1.283 (1.091–1.509)	0.003	1.018 (0.849–1.221)	0.850

Abbreviations: AU, arbitrary units; baPWV, brachial-ankle pulse wave velocity; CAD, coronary artery disease; CI, confidence interval; CRAE, central retinal arteriolar equivalent; SHR, subdistribution hazard ratio. Fine-Gray competing-risk models included all listed covariates. Reflectivity is reported per 10 AU, CRAE per 1 μm, and baPWV per 1 cm/ms. Em dashes indicate that a stable estimate was unavailable because no CAD events occurred in the urinary-protein-positive category.
