# Supplementary material for: Retinal Arterial Wall Reflectivity and Incident Cardiovascular Disease
Source: Ophthalmol Sci. 2026 Sep 4;6(11):101399. doi: 10.1016/j.xops.2026.101399 (PMC13671367; doi:10.1016/j.xops.2026.101399)
Supplement: Supplementary file 6 [file mmc6.pdf]

Supplementary Table 6. Multivariable Fine-Gray Competing Risk Analysis for Cardiovascular Disease in Older Population (n=1,057, age ≥ 70 years)

	Cardiovascular disease (event=61, competing=57)		Stroke (event=28, competing=90)		Coronary artery diseases (event=33, competing=85)	
Variable	SHR (95% CI)	P-value	SHR (95% CI)	P-value	SHR (95% CI)	P-value
Age, per year	1.054 (0.960–1.156)	0.270	1.058 (0.924–1.211)	0.410	1.059 (0.928–1.208)	0.400
Female sex (vs male)	0.462 (0.218–0.979)	0.044	0.253 (0.086–0.745)	0.013	0.827 (0.286–2.391)	0.730
Systolic blood pressure, per 1 mmHg	1.021 (1.007–1.035)	0.002	1.033 (1.009–1.058)	0.007	1.013 (0.997–1.030)	0.110
Non-high-density lipoprotein cholesterol, per 1 mg/dL	0.990 (0.980–0.999)	0.028	0.996 (0.984–1.008)	0.520	0.984 (0.970–0.998)	0.026
High-density lipoprotein cholesterol, per 1 mg/dL	0.990 (0.974–1.006)	0.210	0.996 (0.971–1.022)	0.780	0.984 (0.963–1.005)	0.140
Urinary protein ≥1+ (vs <1+)	4.326 (2.072–9.032)	<0.001	4.586 (1.450–14.505)	0.010	3.876 (1.410–10.655)	0.010
Smoking history (yes vs no)	0.929 (0.481–1.795)	0.830	0.682 (0.285–1.635)	0.390	1.363 (0.509–3.646)	0.540
Diabetes (yes vs no)	1.760 (0.950–3.260)	0.072	0.710 (0.190–2.652)	0.610	2.952 (1.451–6.007)	0.003
No antihypertensive medication use (vs use)	1.089 (0.642–1.848)	0.750	1.875 (0.871–4.037)	0.110	0.643 (0.292–1.417)	0.270
baPWV, per 1 cm/ms	1.619 (1.265–2.073)	<0.001	0.657 (0.201–2.153)	0.490	2.023 (1.668–2.452)	<0.001
CRAE, per 1 μm	0.996 (0.977–1.016)	0.710	0.989 (0.959–1.019)	0.460	1.003 (0.978–1.030)	0.800
Arterial wall reflectivity, per 10 AU	0.875 (0.731–1.047)	0.150	0.836 (0.682–1.024)	0.084	0.930 (0.709–1.220)	0.600

Abbreviations: SHR, subdistribution hazard ratio; CI, confidence interval; baPWV, brachial-ankle pulse wave velocity; AU, arbitrary units. SHRs are reported per 10-AU increase in reflectivity, per 1-μm increase in CRAE, and per 1-cm/ms increase in baPWV. Models adjust for all variables listed. This post hoc subgroup analysis is exploratory.
